# Adverse Drug Reactions and Prescription Patterns of Antiretroviral Drugs: A Longitudinal Observational Study From a Tertiary Care Hospital in Western India

**DOI:** 10.7759/cureus.56424

**Published:** 2024-03-18

**Authors:** Vijaya Dhaarani Sekar, Kavita Joshi, Shruti Bhide, Shirish Rao, Chetan Phirke, Saurabh Patil, Rahul Kothari, Mudra Patel, Arun Shankar

**Affiliations:** 1 Department of Pharmacology and Therapeutics, Seth Gordhandas Sunderdas Medical College and King Edward Memorial Hospital, Mumbai, IND; 2 Department of Internal Medicine, Seth Gordhandas Sunderdas Medical College and King Edward Memorial Hospital, Mumbai, IND; 3 Department of Research, Association for Socially Applicable Research, Pune, IND; 4 Department of Pharmacology, Hinduhridaysamrat Balasaheb Thackeray Medical College and Dr. R. N. Cooper Municipal General Hospital, Mumbai, IND

**Keywords:** antiretroviral (arv), haart therapy, hiv drugs, infectious disease medicine, antiretroviral drug toxicity, pharmacology treatment, drug-related side effects and adverse reactions, antiretroviral drugs, dolutegravir, hiv aids

## Abstract

Background

In 2018, the World Health Organisation (WHO) released interim guidelines, advising a change of regimens to dolutegravir-based first- and second-line antiretroviral therapy (ART), based on which, in 2021, the National Aids Control Organisation (NACO) updated its guidelines to include the tenofovir + lamivudine + dolutegravir (TLD) regimen as a first line of therapy for all people living with HIV (PLHIV) and second- and third-line regimens to dolutegravir-based regimens. Considering this change of regimen, the adverse drug reaction (ADR) profiling and longitudinal prescription pattern of antiretroviral and concomitant medications in adult patients at the ART centre of a tertiary care hospital were assessed in this study.

Methods

Ninety-seven PLHIV out of all the patients who attended the ART centre from September 2021 to July 2022 were enrolled and followed up for six months. The ADRs that occurred during this period were collected along with details of prescription patterns and analyzed by descriptive statistics. Causality assessment for ADR was done using the World Health Organisation-Uppsala Monitoring Centre (WHO-UMC) scale.

Results

Seventy-eight percent (n=76 out of 97) of patients experienced at least one ADR, and 128 ADRs were seen in 97 patients. The most common ADRs were increased alkaline phosphatase (39.0%, n=128), dyslipidaemia (12.5%, n=128), and nephrotoxicity (10.1%, n=128). The drug most suspected of causing ADRs was dolutegravir (27.5%, n=342). The most common therapeutic regimen was TLD (71.2%, n=97). The most prescribed drug was lamivudine (30.6%, n=1183). The most prescribed concomitant medication was cotrimoxazole (15%, n=312).

Conclusions

Dolutegravir-based regimens have been implemented for PLHIV in a phased-out manner from previous non-dolutegravir-based ART regimens, which is in line with the recent NACO guidelines. However, it has also led to an increase in dolutegravir-associated ADRs like increased alkaline phosphatase, dyslipidaemia, and nephrotoxicity. Continuous monitoring of prescriptions and ADRs can add to our knowledge regarding their use and ADRs.

## Introduction

In our world today, with a vaccine for almost every infectious disease, it has been a challenge to develop a vaccine owing to HIV’s unique characteristics in terms of its rapid replication, high levels of mutagenesis, evasion of immune system defences, and the ability to create reservoirs in the host [1]. In addition to this, once a patient is infected, the treatment for the disease is lifelong, causing short- and long-term toxicities, despite all its benefits to the patient. The antiretroviral therapy (ART) programme of India is the second largest programme globally and is acclaimed as one of the best public health programmes providing HIV care services. The goals of ART treatment, as outlined by the National AIDS Control Organisation (NACO), are to improve the quality of life, increase survival clinically, achieve the greatest possible sustained viral load reduction virologically, and facilitate both qualitative and quantitative immune reconstitution immunologically. Direct access to free diagnostic facilities, free first-line therapy, second- and third-line ART, prevention of parent-to-child transmission of HIV (PPTCT) services, prevention, diagnosis, and management of opportunistic infections, including management of TB with daily anti-TB treatment, has been instrumental in bringing down the disease burden and incidence rate of the disease [2].

In 2018, WHO released interim guidelines, advising a change of regimens to dolutegravir-based first- and second-line ART, with a word of caution about the use of dolutegravir in women of childbearing potential about the possibility of neural tube defects [3]. However, in 2019, WHO issued updated guidelines that advise the use of dolutegravir-based regimens as dolutegravir was found to be highly potent, with fewer toxicities and side effects, rapid viral suppression, prevention of resistance, and minimal drug interactions, and also calls for a once-daily dosing regimen [3].

From July 2020, the Technical Research Group advised dolutegravir-based regimens for HIV-positive adults, adolescents, and children (weighing more than 20 kg/age more than six years) under the National Aids Control Programme (NACP) and phased out the transition of patients to dolutegravir-based regimens [4]. In line with this, NACO updated its guidelines to include the tenofovir + lamivudine + dolutegravir (TLD) regimen as the first line of therapy for all people living with HIV (PLHIV) and dolutegravir-based second- and third-line regimens. Most of the data available on the safety of dolutegravir was obtained from controlled, randomised clinical trials, highlighting the importance of conducting observational studies to assess drug safety in clinical practice [5,6].

In light of this change in regimen, it was interesting to study the adverse drug reaction (ADR) profiling and longitudinal prescription pattern of antiretroviral and concomitant medications in adult patients at the ART centre of a tertiary care hospital.

## Materials and methods

Study design

This was an observational, prospective, longitudinal, single-centred study conducted in the Outpatient Antiretroviral Treatment Centre in a tertiary care hospital in Mumbai between September 2021 and July 2022. A total of 97 patients out of all the patients coming to the ART centre of any gender, diagnosed with HIV, above the age of 18, and attending ART clinics who were either ART-naive or experienced patients, including pregnant and lactating women in a stable condition, were included in the study. Only those patients with ADR confirmed based on clinical presentation or laboratory diagnostic parameters were considered for ADR profiling. Patients with serious conditions requiring hospitalisation and already admitted, those who did not consent, those with a history of active substance abuse, and HIV-positive patients who were not currently taking any ART medication were excluded from the study.

Ethical considerations

This study was initiated after obtaining permission from the Institutional Ethics Committee II of Seth Gordhandas Sunderdas Medical College and King Edward Memorial Hospital (approval number: IEC(II)/OUT/531/2021), followed by NACO permission (approval number: MDACS/708/APD). The study was registered with the Clinical Trials Registry of India (CTRI registration number: CTRI/2021/08/035938). The patients were enrolled after giving written informed consent. The anonymity of the patient and confidentiality of the identity of the patient and study data were strictly maintained while obtaining written informed consent and throughout the study.

Sample size and sampling technique

Considering the COVID-19 pandemic and the hesitancy of PLHIV to participate in research studies, a duration-based sampling method with convenience sampling was used, based on which 97 patients were recruited between September 2021 and January 2022.

Study procedure

The patients were enrolled after taking written informed consent, and data was collected prospectively for six months in a structured case record form.

Outcomes

The primary outcome includes the profiling of ADR as reported by patients or confirmed by laboratory and/or clinical findings during the six-month follow-up period. ADR causality with ART drugs was assessed using the World Health Organisation-Uppsala Monitoring Centre (WHO-UMC) scale.

The secondary outcome includes (1) prescription patterns and WHO prescription indicators, which were assessed by the prescribed ART regimens, FDCs, any change in the regimen (with reasons), and use of concomitant medications during the six-month follow-up. (2) Baseline demographic outcomes like age, sex, weight, duration of HIV, and comorbidities were also assessed.

Statistical analysis

Data was entered and analysed using Microsoft Excel (Microsoft Corporation, Redmond, WA, USA). ADR analysis was done as per patient analysis. Prescription analysis was done as per the prescription encounter. All quantitative data are expressed as mean with standard deviation (SD) or median with interquartile range (IQR), whereas all categorical data were analysed using descriptive statistics and expressed as the frequency with percentage.

## Results

Baseline characteristics

A total of 97 PLHIV were enrolled, and 59.8% (58) were males and 40.2% (39) were females. The median age of patients was 47 years (IQR: 42-51), and their median weight was 55 kg (IQR: 48-63). The date of starting ART therapy was available for 73.2% (71) patients, based on which the average duration of disease in these patients was a median of nine years (IQR: 7-14); 37.1% (36 out of 97) patients had comorbidities like hypertension (17 out of 36), diabetes mellitus (nine out of 36), dyslipidaemia (seven out of 36), asthma (two out of 36), and deep vein thrombosis (one out of 36).

ADRs

Seventy-eight point four percent (78.4%, 76 out of 97) of enrolled patients experienced ADRs, of which 44 experienced at least one ADR. A total of 128 ADRs were observed in 76 patients, and the average number of ADRs per patient was 1.6. An account of the number of ADRs seen in the patients is summarised in Table 1.

**Table 1:** Number of ADRs seen in the patients of the prospective arm (n=76) ADRs: adverse drug reactions

Number of ADRs in patients	Number of patients with ADR
1	44
2	14
3	16
4	1
5	1

**Figure 1:**
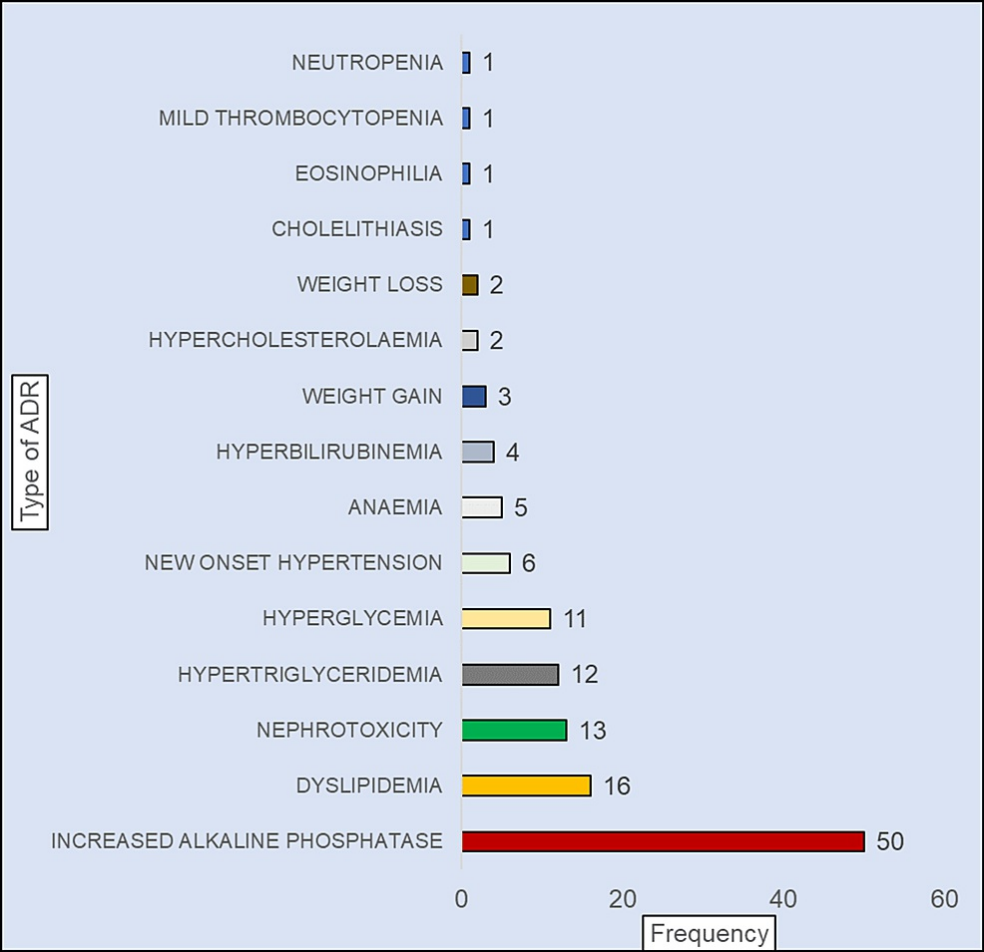
Type of ADR seen with ART medication in the six-month follow-up period (n=128) ADR: adverse drug reaction, ART: antiretroviral therapy

Nine point four percent (9.4%, 12 out of 128) of ADRs were clinical ADRs, whereas 90.6% (116 out of 128) were laboratory ADRs. Figure 1 shows the type and frequency of clinical and laboratory-confirmed ADRs with increased alkaline phosphatase (50 out of 128) followed by dyslipidaemia (16 out of 128) and nephrotoxicity (13 out of 128).

ADRs were observed over a period of six months’ time and reported. During this period, the patients underwent regimen changes as per the latest NACO protocol. At the end of the six-month period, we observed a total of 128 ADRs. Of the 128 reactions, 109 (85.15%) were because of dolutegravir-based regimens, and 19 (14.84%) were because of non-dolutegravir-based regimens. The frequency of various ADRs of dolutegravir-based regimens is given in Table 2, and the frequency of various ADRs of non-dolutegravir-based regimens is given in Table 3.

**Table 2:** Frequency of various ADRs of dolutegravir-based regimens ADR: adverse drug reaction

ADR due to dolutegravir-based regimens	Frequency (n)
Increased alkaline phosphatase	46
Dyslipidaemia	14
Nephrotoxicity	13
Hypertriglyceridemia	10
Hyperglycemia	9
New onset hypertension	6
Anaemia	5
Weight gain	3
Hypercholesterolaemia	2
Weight loss	1
Total number of ADRs	109

**Table 3:** Frequency of various ADRs of non-dolutegravir-based regimens ADR: adverse drug reaction

Non-dolutegravir-based regimens	Frequency (n)
Hyperbilirubinemia	4
Increased alkaline phosphatase	4
Dyslipidaemia	2
Hyperglycemia	2
Hypertriglyceridemia	2
Weight loss	1
Mild thrombocytopenia	1
Cholelithiasis	1
Neutropenia	1
Eosinophilia	1
Total no. of ADRs	19

Table 4 shows suspected drugs responsible for ADRs in the follow-up period with dolutegravir (27.5%) and tenofovir (25.7%).

**Table 4:** Suspected drug responsible for ADR in the prospective (n=342) ADR: adverse drug reaction

Suspected drug	Frequency (%)
Dolutegravir (DTG)	94 (27.5)
Tenofovir (3TDF)	88 (25.7)
Lamivudine (3TC)	82 (24)
Ritonavir (r)	25 (7.3)
Atazanavir (ATV)	16 (4.7)
Abacavir (ABC)	10 (2.9)
Nevirapine (NVP)	9 (2.6)
Zidovudine (AZT)	8 (2.3)
Lopinavir (LPV)	6 (1.8)
Efavirenz (EFV)	4 (1.2)

This study was observational, with the objective of pragmatically assessing the prescriptions and occurrence of ADRs. A risk analysis was carried out at the end of the study, as shown in Table 5.

**Table 5:** 2*2 contingency table for the relative risk of developing an ADR between dolutegravir-based regimens and non-dolutegravir-based regimens ADR: adverse drug reaction

Type of drug regimen	Occurrence of ADR		Total
	Yes	No	
Dolutegravir-based regimen	69 (a)	18 (b)	87 (a+b)
Non-dolutegravir-based regimen	7 (c)	3 (d)	10 (c+d)
Total	76 (a+c)	21 (b+d)	

The relative risk for a patient to develop an ADR on a dolutegravir-based regimen was 1.133, and an increased risk of getting an ADR by 0.133 times was seen in patients taking dolutegravir-based regimens. According to the causality assessment as per the WHO UMC scale, out of the total of 342 drugs responsible for ADRs, 206 (60.2%) were possible, and 136 (39.8%) drugs were probable for the causality of ADRs.

Prescription patterns and WHO prescription indicators

Participants had a total of 383 prescription encounters during the study time period resulting in an average of 3.9 prescriptions per patient during the six-month follow-up period. Five patients were lost to follow-up: one at the two-month visit, one at the four-month visit, and three at the six-month visit. The WHO prescription indicators are summarised in Table 6.

**Table 6:** WHO prescription indicators for ART drugs and regimes ART: antiretroviral therapy, SD: standard deviation, FDC: fixed-dose combination

Indicator	Value
Number of patients	97
Total number of prescription encounters	383
Total number of ART medications	1183
Average no of ART Medications per prescription ± SD	3.08 ± 0.298 (95% CI, 2.78-3.37)
Total number of concomitant medications	312
Average no of concomitant medications per prescription ± SD	0.814 ± 0.076 (95% CI, 0.737-0.89)
Percentage of ART drugs prescribed by generic name	100%
No. of ART FDCs	396
No. of single drugs	49

The most prescribed ART regimen of the 383 therapeutic regimens prescribed was TLD (71.28%), followed by tenofovir + lamivudine + atazanavir + ritonavir (5.48%) (Table 7).

**Table 7:** Frequency distribution of various ART regimens in prescriptions (n=383) TLD: tenofovir + lamivudine + dolutegravir TL+ATV/r: tenofovir + lamivudine + atazanavir boosted with ritonavir ZLN: zidovudine + lamivudine + nevirapine ABC/L+DTG: abacavir + lamivudine + dolutegravir LPV/r+DTG: lopinavir boosted with ritonavir + dolutegravir ZL+ATV/r: zidovudine + lamivudine + atazanavir boosted with ritonavir DRV/r+DTG: darunavir boosted with ritonavir + dolutegravir ABC/L+EFV: abacavir + lamivudine + efavirenz TLE: tenofovir + lamivudine + efavirenz TL+NVP: tenofovir + lamivudine + nevirapine ZL+EFV: zidovudine + lamivudine + efavirenz ABC/L+LPV/r: abacavir + lamivudine + lopinavir boosted with ritonavir ART: antiretroviral therapy

Regimen	Frequency (percentage %)
TLD	273 (71.28)
TL+ATV/r	21 (5.48)
ZLN	21 (5.48)
ABC/L+DTG	17 (4.44)
LPV/r+DTG	12 (3.13)
ZL+ATV/r	12 (3.13)
DRV/r+DTG	8 (2.09)
ABC/L+EFV	6 (1.57)
TLE	6 (1.57)
TL+NVP	4 (1.04)
ZL+EFV	2 (0.52)
ABC/L+LPV/r	1 (0.26)

The most prescribed antiretroviral drug out of a total of 1183 drugs was lamivudine (30.6%), followed by dolutegravir (26.2%) and tenofovir (25.6%) (Table 8). Lastly, cotrimoxazole (15.06%) was the most prescribed concomitant medication, followed by amlodipine (13.14%) (Table 9).

**Table 8:** Frequency distribution of ART drugs in prescriptions (n=1183) ART: antiretroviral therapy

ART drug	Frequency (percentage %)
Lamivudine	363 (30.68)
Dolutegravir	310 (26.20)
Tenofovir	304 (25.70)
Ritonavir	54 (4.56)
Zidovudine	35 (2.96)
Atazanavir	33 (2.79)
Nevirapine	25 (2.11)
Abacavir	24 (2.03)
Efavirenz	14 (1.18)
Lopinavir	13 (1.10)
Darunavir	8 (0.68)

**Table 9:** Frequency distribution of concomitant medications in prescriptions (n=312)

Concomitant medication	Frequency (percentage %)
Cotrimoxazole	47 (15.06)
Amlodipine	41 (13.14)
Atorvastatin	32 (10.26)
Metformin	32 (10.26)
Telmisartan	30 (9.62)
Glimipiride	20 (6.41)
Isoniazid	16 (5.13)
Pyridoxine	16 (5.13)
Levocetirizine	14 (4.49)
Losartan	12 (3.85)
Atenolol	8 (2.56)
Chlorthalidone	8 (2.56)
Ecospirin	8 (2.56)
Gliclazide	8 (2.56)
Nifedipine	8 (2.56)
Apremilast	4 (1.28)
Rivaroxaban	4 (1.28)
Vildagliptin	4 (1.28)

Change of regimen

During the six-month follow-up period, 25% (24 out of 97) patients had a change of regimen. All these 24 patients were changed into dolutegravir-based regimens from non-dolutegravir-based regimens, according to new guidelines by substitution. Eighteen out of 24 patients were put on the TLD regimen, and six out of 24 were put on the abacavir + lamivudine + dolutegravir regimen.

## Discussion

The TLD regimen was the most prescribed regimen observed in this study. A shift to dolutegravir-based regimens like TLD, ABC/L + DTG, and LPV/r + DTG from previous regimens as per the latest WHO and NACO guidelines was observed. The ADRs were mainly laboratory findings like increased alkaline phosphatase, hyperglycemia, and dyslipidaemia. The relative risk for a patient to develop an ADR on a dolutegravir-based regimen was 1.133, and an increased risk of getting an ADR by 0.133 times was seen in patients taking dolutegravir-based regimens. The most common drugs and regimens responsible for ADRs were dolutegravir and the TLD regimen, respectively.

The average number of ART drugs per prescription encounter was 3.08 ± 0.298 (95% CI, 2.78-3.37 ). Parmar et al. also reported that the average number of drugs per encounter was 3.96 [7]; however, in this study, both ART medications and concomitant medications were included to calculate the average number of drugs per encounter. Lamivudine being the most prescribed drug is in concordance with the study published by Parmar et al. and in line with NACO guidelines stating that NRTIs like lamivudine should be used as the backbone of ART regimens [7,8]. Our finding that the TLD regimen is most commonly prescribed is in contrast to the results of studies conducted by Sonali et al. [9] in the year 2020, when old guidelines were in place, where the tenofovir + lamivudine + efavirenz regimen was the most commonly prescribed regimen (69.9%), followed by zidovudine + lamivudine + nevirapine (19%). As per the new guidelines for implementation of dolutegravir-based regimens for first- and second-line treatments by NACO and WHO from 2020 and 2019, respectively, which were implemented at the beginning of our study, we found the phase-wise change to dolutegravir-based regimens [4,8]. Cotrimoxazole was found to be the most commonly prescribed concomitant drug, which is consistent with the study conducted by Parmar et al., which found that cotrimoxazole was prescribed in 10.1% of prescription encounters [7].

More than two-thirds of patients experienced ADRs. Interestingly, in this study, we found that 91.7% of these ADRs were laboratory ADRs. This is in contrast to the studies that have reported a higher number of patient-reported ADRs like hypersensitivity reactions (54.7%) and gastritis (12.6%) by Murthy et al. [10] and Sadiq et al. [11], respectively. Previous studies, like the study conducted by Rukmangathen et al. [12] and Murthy et al. [10], reported laboratory ADRs, such as anemia (222.17% and 31.5%, respectively, in patients receiving zidovudine + lamivudine + nevirapine regimens or tenofovir + lamivudine + efavirenz regimens. A review of dolutegravir by Kandel et al. [13] stated that the most consistently seen ADR with dolutegravir was raised creatine levels and a mild elevation of liver transaminases (5%). It also states that the frequency of ADRs occurring in patients was minimal in comparison to other protease inhibitor-based regimens [13,14]. In our study, we report a broader range of laboratory ADRs, like increased alkaline phosphatase, dyslipidaemia, and nephrotoxicity. These laboratory ADRs are consistent with the side effect profile of dolutegravir-based regimens as established by studies and FDA labels for dolutegravir [5,6,14]. However, the reduced number of patients who reported ADRs in our study could be attributed to the lower side effect profile claimed by studies conducted by Phillips et al. [15].

According to the WHO-UMC causality assessment, 60.2% were established to have a possible association. We report that the causality assessment may have limitations as the association of ADRs to individual drugs was not possible as almost all drugs were given as fixed drug combinations or in combination with other antiretroviral drugs. Also, de-challenge and rechallenge were not done in these cases to establish certainty due to ethical challenges. However, the results of the WHO-UMC causality assessment are similar to those of the study conducted by Murthy et al., where they had a possible association [10], and Jain et al., where they were able to establish 72.4% as a possible association [16].

A change in regimen under NACO guidelines occurs primarily in two categories. Either a patient is switched to another regimen or substituted for another regimen as per guidelines. Switching of a regimen occurs exclusively under two categories: immunological failure or virological failure, where an identified drug or regimen is usually switched out to another drug or regimen. Substitution occurs in cases of toxicity or side effects, drug-drug interactions, and/or in cases of policy changes to the existing guidelines [2,4]. The results of the most prescribed regimen, drug, ADR profile, and newly prescribed regimens could all be attributed to the change of guidelines that were implemented during the beginning of this study and are well reflected in the study results as well.

Strength, limitations, and future directions

This could be one of the first studies conducted after the change in NACO guidelines in 2020 that assesses prescription patterns, NACO guidelines implementation, and associated ADRs. The prospective longitudinal nature of the study also enhances the quality of evidence generated through the study. However, the results of the study must be interpreted in view of limitations like small sample sizes and single-centre samples. Also, the causality of ADRs needs to be interpreted with caution as many ART drugs are administered as fixed-dose combinations, which interferes with the WHO UMC scale of assessing causality. The implementation of dolutegravir-based regimens by NACO is still ongoing, and we captured the crucial initial part. Continuous monitoring of prescriptions and ADRs can add to our knowledge regarding their use and ADRs.

## Conclusions

Dolutegravir-based regimens have been implemented for PLHIV in a phased-out manner from previous non-dolutegravir-based ART regimens, which is in line with the recent NACO guidelines of 2020, for the majority of the patients in our study. However, it has also led to an increase in ADRs like increased alkaline phosphatase, dyslipidaemia, and nephrotoxicity associated with dolutegravir and dolutegravir-based therapeutic regimens in comparison to non-dolutegravir-based therapeutic regimens. Continuous monitoring of prescriptions and ADRs with long-term follow-up can add to our knowledge regarding their use and ADRs.
